# Correction: Kernel methods and their derivatives: Concept and perspectives for the earth system sciences

**DOI:** 10.1371/journal.pone.0246775

**Published:** 2021-02-03

**Authors:** J. Emmanuel Johnson, Valero Laparra, Adrián Pérez-Suay, Miguel D. Mahecha, Gustau Camps-Valls

The affiliation for the fourth author is incorrect. The correct affiliations are not indicated. Miguel D. Mahecha is not affiliated with #1 but with: Remote Sensing Centre for Earth System Research, Leipzig University, Germany and Helmholtz Centre for Environmental Research, UFZ, Leipzig Germany.
