# Supplementary material for: Kernel methods and their derivatives: Concept and perspectives for the earth system sciences
Source: PLoS One. 2020 Oct 29;15(10):e0235885. doi: 10.1371/journal.pone.0235885 (PMC7595302; doi:10.1371/journal.pone.0235885)
Supplement: Attachment — Submitted filename: sakame_rebuttal_letter.pdf [file pone.0235885.s001.pdf]

VNIVERSITAT DE VALÈNCIA

Image Processing Laboratory (IPL)
Parc Científic Universitat de València – Campus Universitario de Paterna
UNIVERSITAT DE VALÈNCIA
E- 46980 Paterna (Valencia), SPAIN
Telephone: +34 627 61 00 84 | Fax: +34 963 54 40 56

7th July, 2019

Responses to reviewers

Reviewer I

1. The topic of the paper is interesting, however it is not clear to me what is the contribution of this paper.

   In order to address reviewer's comment, we included the following sentences that highlight our contributions in the corresponding sections of the new version of the manuscript.

   ***In the Paper**:*
   - *The aim of this work is to show that it is indeed possible to interpret the functions learned by various kernel methods in a much more intuitively that commonly assumed. Specifically, we show that despite their complexity, derivatives of these functions have a simple mathematical formulation, are easy to compute, and can be applied to many different problems. This goes in line, and tries to elevate the debate, of understanding rather than just fitting in the context of kernel machines.*
   - *We provide the explicit analytic form of the first and second derivatives of the most common kernel functions with regard to the inputs as well as generic formulas to compute higher order derivatives.*
   - *We suggest summarizing derivatives in the form of sensitivity measures, either point-wise or dimension-wise, to study the relevant points and features for the learned model. Derivatives of the function are indeed useful to compute explicit measures of sensitivity of the learned function, and hence to study models easily, either for classification, regression, density or dependence estimation.*
   - *We provide empirical evidence of the usefulness of the derivatives in kernel machines in both illustrative toy examples, and a set of real examples in the context of Earth system science.*

2. I believe the computation of the derivatives of different models and kernel functions is not new.

In this manuscript, our intention was not to give the impression that we introduce derivative of the kernel is our contribution. We were more focused on connecting the notation that the derivative of a kernel can be seen as a unified framework with potential applications to kernel methods in general. We synthesize the literature which spans over many different disciplines and unified the concept. Our hope is that future researchers will be able to look at our paper as a reference or summary of the literature with the ideas well explained and connected with riveting toy examples and applied data explorations.

3. One of the motivations of this work, as stated in the Abstract and in the Introduction, is to give insights about the learned functions. However, I think the paper fails in providing such insights.

   As mentioned in the above section about the contributions, we have removed the phrase 'gives insights about the learned functions' as it is unclear and misrepresents our intentions and contributions. Thanks for the remark. We have replaced it with "The aim of this work is to show that it is indeed possible to interpret the functions learned by various kernel methods in a much more intuitively that commonly assumed." We firmly believe that this better captures our intentions.

4. Beginning of page 3: authors state that the derivatives can be related to the margin. The closest assessment of this sentence is in Section 3.3 and the corresponding Figure 2. The figure is a little unclear (authors don't clarify what is the y axis in the plots, I this it should be the norm of the learnt model W). In fact, the norm of W is related to the margin, but the paper doesn't clarify this.

   - ***4.2 Function Derivatives and Margin***
     This has been fixed.
   - **The y-axis in the plot**
     This has been fixed.
   - **The relation between the norm of W and the margin**
     We have clarified the point about W in section 3.3. The ||W|| was misleading as it really should have been ||f||. The regularization term ||.|| was chosen to be different for the experiment done in section 3.3. The relation specifically with the margin is mentioned in the following section with the support vector machines (4.2).

     We want to thank the reviewer for pointing this issues.

5. I appreciate experiments on toy data for gaining insights on the theory. However, the aim of the proposed analysis is to gain insights on the learnt models, from real data, where such insights are actually needed. For instance, the plots in Fig. 2 can be computed on real datasets, and the generalization performance of the learnt models varying the regularization can be assessed.

   Thanks for the comment. In the new version of the paper we include a full new section showing the application of the concepts of derivatives and sensitivity of kernel methods in Earth system spatial-temporal data. Today data-driven research in Earth system dynamics has gained momentum and complement global modelling efforts. Much of Earth data is generated by a wide range of satellite sensors, upscaled products from *in-situ* observations, and model simulations with constantly improved space and time resolutions. We illustrate the use of the kernel derivatives in Gaussian processes, principal curve estimates and dependence estimation in three selected spatio-temporal Earth observation variables of interest. We will pay attention to how using kernel derivatives may help in (1) choosing the appropriate space and time scales to analyze phenomena, (2) visualize the most informative areas of interest, and (3) detect anomalies in spatio-temporal Earth data. We agree in that this effort was needed, and the set of experiments illustrate the strong capabilities of our proposal to deal with Earth monitoring and learning from kernel learning models. Thanks for the suggestion that we took very seriously.

6. Section 6.4 looks also interesting, but it is very short and not clear enough.

   We want to thank the reviewer for this comment. We improved the section including comments and clarifications in two ways. First, by clarifying that the derivatives of the, e.g. HSIC, dependence function are actually related to the concept of *leverage* in statistics. Recently, this notion has been introduced in the context of [Alaoui, 2015]. And secondly, by pointing out that HSIC can be understood as a maximum mean discrepancy (MMD) [Gretton, 2012] between the joint probability measure of the involved variables and the product of their marginals, and MMD derivatives are very similar to those of HSIC provided here. The explicit use of the kernel derivatives would allow us to use gradient-descent approaches in methods that take advantage of HSIC or MMD, such as in algorithms for domain adaptation and generative modeling.

I think the kind of analysis proposed by the paper is interesting, however there are some major shortcomings:
-The paper contribution is not clear and it is missing experiments on real-world datasets, that should assess if the proposed analysis may be applicable and useful to real data.

As mentioned above, we agree that the previous version of the paper was not clear in identifying the contributions clearly. We have rectified this aspect in the new version. The main contributions are:

- *To show that it is indeed possible to interpret the functions learned by various kernel methods have derivatives with simple mathematical formulations, are easy to compute, and can be applied to many different problems.*
- *To provide the explicit analytic form of the first and second derivatives of the most common kernel functions with regard to the inputs as well as generic formulas to compute higher order derivatives.*
- *To provide illustrative toy examples with similar formulations and suggest some summarizing aspects of derivatives (i.e. sensitivity)*
- *To have included an entire section for problems related to Earth system data. Each problem corresponds to the toy examples illustrated and we provide empirical evidence of the usefulness of the derivatives in kernel machines in the context of Earth system science.*

References

- Alaoui, Ahmed and Mahoney, Michael W, "Fast Randomized Kernel Ridge Regression with Statistical Guarantees", *Advances in Neural Information Processing Systems 28*, (775-783), 2015
- Gretton, Arthur and Borgwardt, Karsten M. and Rasch, Malte J. and Schölkopf, Bernhard and Smola, Alexander, "A Kernel Two-sample Test", *Journal of Machine Learning Research*, (723-773), 2012
