# Supplementary material for: Kernel methods and their derivatives: Concept and perspectives for the earth system sciences
Source: PLoS One. 2020 Oct 29;15(10):e0235885. doi: 10.1371/journal.pone.0235885 (PMC7595302; doi:10.1371/journal.pone.0235885)
Supplement: Attachment — Submitted filename: SAKAME - 2020 - Editor Comments.pdf [file pone.0235885.s002.pdf]

Image Processing Laboratory (IPL)
Parc Científic Universitat de València – Campus Universitario de Paterna
UNIVERSITAT DE VALÈNCIA
E- 46980 Paterna (Valencia), SPAIN
Telephone: +34 627 61 00 84 | Fax: +34 963 54 40 56

5th April, 2020

Dear Editor,

We received your comments for our submitted manuscript to PLOS One (PONE-D-19-17584). We would like to address them as follows:

We note that your manuscript is not formatted using one of PLOS ONE's accepted file types. Please reattach your manuscript as one of the following file types: .doc, .docx, .rtf, or .tex (accompanied by a .pdf).

We have included the .tex file of the main document and we have also provided the .zip file which has all of the sub .tex files as well as figures.

Please explain the rationale for the development of your approach in light of recent research in this area, clearly indicating which problem with existing methods you are addressing.

We have indicated our intentions and our proposed approaches in (1) The introduction (by giving an overview of our rationale as a whole); (2) Sections 3-6 (which provide some rationale with respect to each of the methods individually as we relate the kernel derivatives and how known the derivatives can provide additional information about each kernel method, plus a summarizing table of the contributions and perspectives) and (3)The intro to section 7 (which gives our rationale for the use in the Earth science data discussing the current state-of-the-art and our proposal).

Please clearly report at the beginning of your methods or results section which the key performance measures were to establish validity and utility of your method. Please also report clearly which statistical analysis was used to establish robustness of performance measures.

We have included an annotation which describes the metrics we used to establish our performance measures in accuracy and robustness for the supervised methods in sections 7.2 and 7.3, and consistently use them through the manuscript.

Please note that PLOS ONE requires that experiments, statistics, and other analyses must be performed to a high technical standard and described in sufficient detail to allow for reproducibility of the study. To demonstrate the performance of the method, we would expect comparisons to be drawn between existing state-of-the-art methods.

In all experiments, we compare the derivative approaches with canonical approaches based on kernels (GP, SVM, KDE and HSIC)), their predictive functions and their intuition. We also give an explanation of the current literature in the beginning of section

7, and have included the code for the interested readers and to ensure reproducibility of the experiments.

Please ensure that you refer to Figure 3 in your text as, if accepted, production will need this reference to link the reader to the figure.

We have revised the paper and have noted the following Figure 3 were referenced in line 282.

Regards,

Juan Emmanuel Johnson (on behalf of the authors)
